# Analysis of the Relative Importance of Stand Structure and Site Conditions for the Productivity, Species Diversity, and Carbon Sequestration of *Cunninghamia lanceolata* and *Phoebe bournei* Mixed Forest

**DOI:** 10.3390/plants12081633

**Published:** 2023-04-12

**Authors:** Yiru Wang, Zhaohua Liu, Tao Tang, Jiping Li

**Affiliations:** 1 Faculty of Forestry, Central South University of Forestry and Technology, Changsha 410004, China; 2 Key Laboratory of State Forestry Administration on Forest Resources Management and Monitoring in Southern Area, Changsha 410004, China; 3 Research Centre of Forest Remote Sensing and Information Engineering, Central South University of Forestry and Technology, Changsha 410004, China

**Keywords:** stand structure, site conditions, forest functions, structural equation model, *Cunninghamia lanceolata*, *Phoebe bournei*

## Abstract

Forest stand structure (the characteristics and interrelationships of live trees) and site conditions (the physical and environmental characteristics of a specific location) have been linked to forest regeneration, nutrient cycling, wildlife habitat, and climate regulation. While the effects of stand structure (i.e., spatial and non-spatial) and site conditions on the single function of *Cunninghamia lanceolata* and *Phoebe bournei* (*CLPB*) mixed forest have been studied in previous studies, the relative importance of stand structure and site conditions in terms of productivity, species diversity, and carbon sequestration remains unresolved. In this study, a structural equation model (SEM) was adopted to analyze the relative importance of stand structure and site conditions for the forest productivity, species diversity, and carbon sequestration of *CLPB* mixed forest in Jindong Forestry in Hunan Province. Our research demonstrates that site conditions have a greater influence on forest functions than stand structure, and that non-spatial structures have a greater overall impact on forest functions than spatial structures. Specifically, the intensity of the influence of site conditions and non-spatial structure on functions is greatest for productivity, followed by carbon sequestration and species diversity. In contrast, the intensity of the influence of spatial structure on functions is greatest for carbon sequestration, followed by species diversity and productivity. These findings provide valuable insights for the management of *CLPB* mixed forest in Jindong Forestry and have significant reference value for the close-to-natural forest management (CTNFM) of pure *Cunninghamia lanceolata* forests.

## 1. Introduction

Forest ecosystems play a vital role in timber production, biodiversity preservation, and carbon sequestration [1,2,3]. Forests can be separated into mixed forests and pure forests according to the number and volume of tree species. Compared to pure forests, mixed forests contain a greater diversity of tree species and a more complex forest structure, allowing them to serve a greater range of forest ecological functions [4,5]. In this context, forest managers are increasingly accepting of close-to-natural forest management (CTNFM) practices. CTNFM, a production system based on the principles of multifunctional forest management, advocates for mixed uneven forests as an ecologically more stable alternative to uniform monocultures [6,7,8]. Therefore, transforming pure plantations into mixed forests by CTNFM is an effective way to improve ecological functions, which is of vital significance for the implementation of China’s forest quality precision enhancement project.

The pure Chinese fir (*Cunninghamia lanceolata*) forests account for the largest proportion of the area of plantations in southern China. Transforming pure Chinese fir forests into mixed forests through CTNFM is an important challenge, aimed at improving the quality of Chinese fir forests. In recent years, a mixed forest management model of *Cunninghamia lanceolata* and *Phoebe bournei* (*CLPB*) has been recognized as the most successful CTNFM method for pure Chinese fir forests. In this model, the rare and valuable broad-leaved species *Phoebe bournei* has been replanted artificially in pure *Cunninghamia lanceolata* plantations to establish *CLPB* mixed forests, which can effectively promote the growth of both trees and improve the ecological function of pure *Cunninghamia lanceolata* plantations [9,10]. In addition, the State Forestry Administration of China has pushed the *CLPB* mixed forest management model as one of the most successful methods for enhancing the quality of Chinese fir forests in southern China.

The stand structure of forests, which encompasses the characteristics and interrelationships of live trees, is a fundamental attribute of forest ecosystems [11]. A well-designed stand structure has important implications for forest ecosystems, including the provision of wildlife habitat, carbon storage for climate regulation, and effective forest regeneration. By optimizing the structure of the stand, the maximum functional benefits of the forest ecological system can be realized [12,13,14,15,16,17]. The forest stand structure can be subdivided into spatial and non-spatial structures. Spatial structure pertains to the arrangement and interrelationships of living tree features and is typically measured using indicators such as mixing degree, angular scale, and size ratio. Non-spatial structure, on the other hand, characterizes the quality of individual trees and is often gauged by factors such as DBH, tree height, and density [18,19,20,21]. Nevertheless, even within a single tree species, growth rates can fluctuate significantly across different stands due to varying forest site conditions, which encompass the physical, chemical, and biological attributes of a specific location such as soil characteristics, topography, and vegetation. Site conditions may thus be one of the important factors that affect forest functions [22,23].

Jindong Forest Farm is located in the south of Hunan Province, which is the main planting area of *Cunninghamia lanceolata* in the Province. In recent years, with the increasing attention paid by the Chinese government to the ecological functions of forests, the forest management objectives of Jindong Forest Farm have shifted from producing wood to enhancing the ecological functions of forests. Hence, converting a vast tract of pure Chinese fir plantation into a mixed forest with enhanced biological functions is one of the greatest challenges facing forest management at Jindong Forest Farm. Through repeated studies on the model of mixing Chinese fir with other needles and broadleaves, Jindong Forest Farm discovered *CLPB* as the most effective CTNFM model for Chinese fir forest. It has thus become a national demonstration site for the *CLPB* mixed forest model [24,25]. Although previous studies have explored the effects of stand structure and site conditions on the single forest function of *CLPB* mixed forest in Jindong Forest Farm [26,27,28,29,30], the relative importance of stand structure and site conditions in terms of productivity, species diversity, and carbon sequestration remain unresolved.

The aim of this study was to explore the relative importance of the stand structure and site conditions of *CLPB* mixed forest to productivity, species diversity and carbon sequestration. To achieve this goal, we have adopted a structural equation model (SEM) approach, which can provide insights into the relative importance of various aspects of stand structure and site condition on forest functions [31,32,33,34]. The following hypotheses were tested: (1) site condition has a greater influence on forest functions (productivity, species diversity and carbon sequestration) than stand structure. (2) The total impact intensity of spatial and non-spatial structures on forest functions is equal.

## 2. Results

### 2.1. Response between Observed Variables

**Figure 1:**
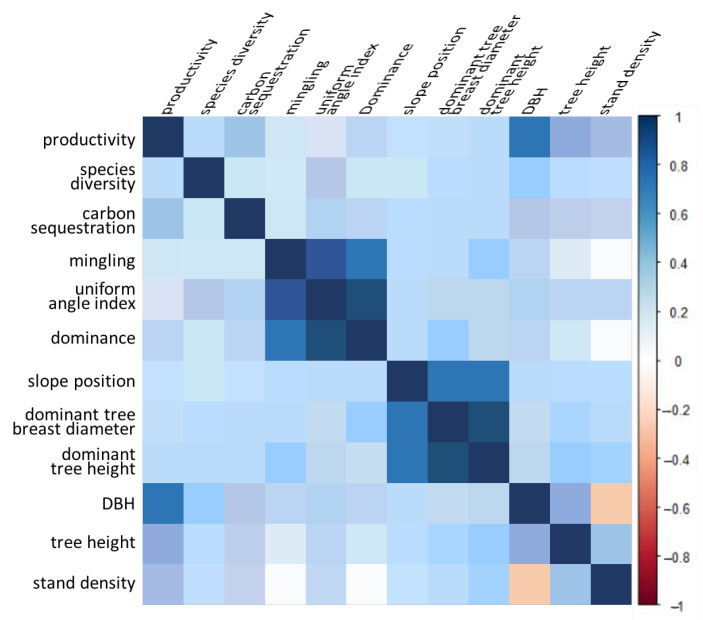
Correlation heat map between structure variables and function variables.

According to the correlation heat map (Figure 1), there is a very significant correlation between the slope position, dominant tree breast diameter and dominant tree height. The slope position has a significant correlation with DBH, and the dominant tree breast diameter is significantly related to the dominant tree height. The DBH is significantly negatively correlated with the stand density. The negative correlation between stand density and DBH is mainly due to the growth factors of the forest trees themselves and competition. Within a certain range, stand density will promote the growth of DBH, and beyond this range, the growth of DBH is inhibited. The DBH, tree height, stand density and stand productivity demonstrate a very significant correlation. Higher tree height and DBH usually mean higher biomass accumulation and higher productivity. In addition, higher density may also have an effect on productivity, but its effect may vary depending on environmental conditions and stand type.

The dominance, mingling and uniform angle index are very significantly correlated. The influence of stand structure indicators on the carbon sequestration function is mainly through aspects such as biomass and tree age. For example, higher tree height and DBH usually imply higher biomass accumulation and higher carbon storage capacity. Due to the conversion relationship between carbon sequestration and productivity, there is a highly significant correlation between them. Forest species diversity is influenced by many factors, and our analysis found a significant correlation between forest species diversity and DBH. Among the possible reasons, higher tree height and DBH may provide more micro-environmental variation in habitat and thus support the presence of more species.

It should be noted that the correlation between stand structure, stand environment and forest productivity, species diversity and carbon sequestration functions is complex and may be influenced by many other factors, such as climate and soil. Therefore, more comprehensive studies and analyses are needed to more accurately assess the correlations between them.

### 2.2. Path Diagram and Standardized Coefficients in the SEM Analysis

**Figure 2:**
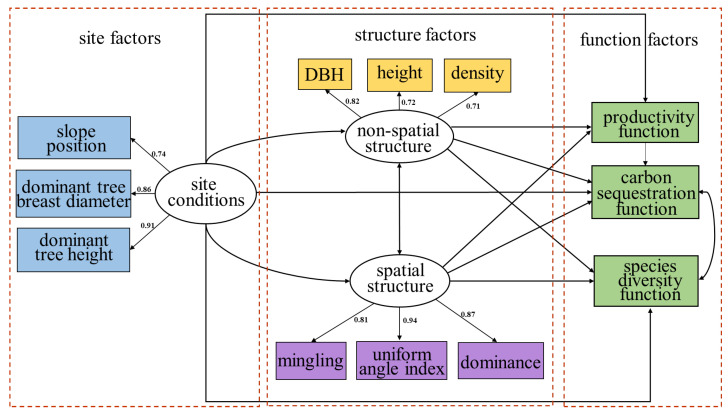
The optimal SEM between stand structures, site conditions and forest functions. The slope position, dominant tree breast diameter and dominant tree height are the explicit variables of site conditions. The DBH, height and density are the explicit variables of non-spatial structure. The mingling, uniform angle index and dominance are the explicit variables of spatial structure.

The test results (Table 1) showed that the RMSEA parameter value of the initial model was 0.055, indicating that the model did not adequately fit the observed data, so further modification of the initial model was required. We improved the previous hypothesis, supplemented the relationship between forest species diversity and carbon sequestration, and reconstructed the path map (Figure 2). After calculating the fitting index of the model and comparing this with the detection standards, the Chi-square degrees of freedom ratio of the optimal model was 1.889, which between 1 and 3, indicating a good fit. RMSEA was less than 0.05, which met the evaluation criteria. It is assumed that the optimal model is adapted to the observation data, and that the test indicators of each adaptation statistic have reached the evaluation standard, indicating that the hypothesis model is well adapted to the data [35].

**Figure 3:**
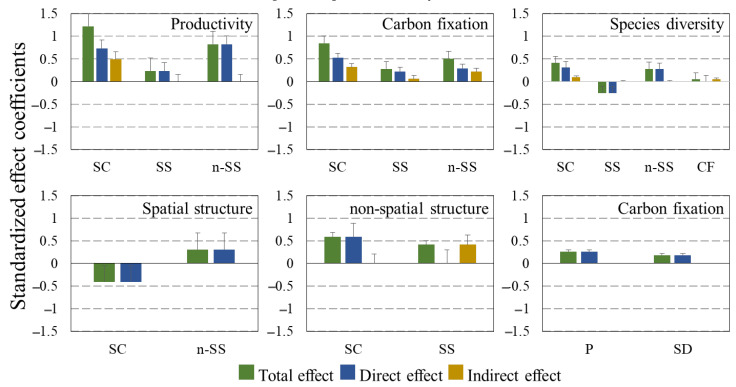
Standardized total, direct and indirect effects of site conditions, spatial structure and non-spatial structure on forest functions. (SC is site conditions, SS is spatial structure, n-SS is non-spatial structure, CF is carbon fixation, P is productivity, SD is species diversity.)

The SEM showed that site conditions had strongly positive effects on the forest productivity function, carbon sequestration function and species diversity function, and the standardized total effects were 1.221, 0.850 and 0.413, respectively (Figure 3). Site conditions also had an indirect influence on productivity, carbon sequestration and species diversity, and these indirect effects were 0.490, 0.321 and 0.102. Comparing stand structure with site conditions, the site conditions had the largest total impact on forest multi-functionality; the coefficient is 2.484, indicating that the forest functions are mainly affected by site conditions. Site conditions had direct and negative effects on spatial structure (−0.409) and direct and positive effects on non-spatial structure (0.593).

There are interactions between spatial structure and non-spatial structure. Spatial structure had indirect and positive effects on non-spatial structure (0.417), while non-spatial structure had direct and positive effects on spatial structure (0.309). The total impact of spatial structure on forest functions (0.267) is less than that of non-spatial structure (1.615). Spatial structure had direct and positive effects on productivity (0.234) and carbon sequestration (0.220), direct and negative effects on species diversity (−0.249), and indirect and positive effects on carbon sequestration (0.062). Non-spatial structure had direct and positive effects on productivity (0.826), species diversity (0.282), and carbon sequestration (0.290), and indirect and positive effects on carbon sequestration (0.217).

There are also interactions between the multiple forest functions. Forest productivity had direct and positive effects on carbon sequestration (0.263). Plant species diversity had a direct impact on carbon sequestration (0.182). Meanwhile, forest carbon sequestration had an indirect impact on species diversity (0.053).

### 2.3. Multi-Factors Analysis of Stand Structures, Site Conditions and Forest Functions

Factors of stand structure and site condition can be quantitatively analyzed using some indicators that can be directly measured, and the degree of response is different. The total effect coefficients of the site conditions on the forest non-spatial structure and on the spatial structure are 0.593 and −0.409, respectively, that is, when the site conditions change by 1, the forest non-spatial structure changes by 0.593 and the forest spatial structure changes by −0.409. The non-spatial structure of forest stands has a correlation coefficient of 0.82 for the breast diameter and 0.72 for the tree height, indicating that changes in the non-spatial structure of the forest stands are more likely to affect the DBH [36].

We found that in the current phase, forest productivity was most closely related to site conditions, not tree size (that is, non-spatial structure, DBH and height). At present, the mixed forest of *CLPB* in this study is in the stage of middle-aged and near mature forest, and the influence of tree size (DBH and tree height) on growth is weaker than that of site conditions. That is, the fertile soil and the slope position of the trees played a significant role in the growth of the forest.

Through the analysis of the SEM, we found that plant species diversity had a direct impact on carbon sequestration (0.182). Meanwhile, forest carbon sequestration had an indirect impact on species diversity (0.053). There was a direct relationship between carbon sequestration and productivity, Moreover, carbon sequestration was weakly correlated with species diversity, while species diversity and productivity were not found to be direct related [37,38]. The relationship between species diversity and productivity is affected by species richness, stand type and environmental heterogeneity, and these influencing factors do not exist independently but rather interact with each other [39,40,41]. Therefore, when studying the relationship between stand functions, it is worth considering the factors that may affect the relationship between them.

## 3. Discussion

The first hypothesis was strongly supported by our results, which showed that site condition has a greater influence on forest functions of *CLPB* mixed forest than stand structure. Figure 1 indicates that forest functions are directly influenced by site conditions; the intensity of the influence in greatest for productivity, followed by carbon sequestration and species diversity. These findings suggest that environmental factors have a more substantial direct impact on productivity than species diversity and carbon sequestration, indicating that productivity exhibits a more pronounced response to site conditions, which is consistent with Liu’s [42] conclusions on the effects of environmental and stand structure factors on productivity. Improvements in site conditions were found to have a more significant impact on forest functions [43].

Our research demonstrates that non-spatial structures have a greater overall impact on forest functions than spatial structures, which was not exactly the same as our second hypothesis. The non-spatial structure has a direct positive effect on forest functions; the intensity of the influence is greatest for productivity, followed by carbon sequestration and species diversity. Forest spatial structure also has a direct effect on forest functions; the intensity of the influence is greatest for carbon sequestration, followed by species diversity and productivity. In addition to the direct effects, stand structure also has an indirect effect on carbon sequestration; the non-spatial structure has a stronger effect than the spatial structure. The indirect impact of the stand structure on productivity and species diversity is zero, and thus, the direct impact coefficient is equal to the total impact coefficient.

The DBH responds more to the non-spatial structure compared to tree height. Geir et al. also found in their study on the relationship between stand density and DBH and tree height that the response of DBH to changes in stand density is greater than that for tree height, which is consistent with the findings of our study [44]. Among the three quantitative indicators of spatial structure, the uniform angle index has the largest response to spatial structure, followed by dominance and then mingling. This is because the mixed forest of *CLPB* in our study has low tree species diversity. Most studies have reported a positive relationship between tree growth and the degree of mixing. Zhang discovered that in stands with greater tree species diversity, the effect of mingling on the stand growth would be enhanced, and the importance of mingling on the spatial structure would increase [45].

## 4. Materials and Methods

### 4.1. Study Site

**Figure 4:**
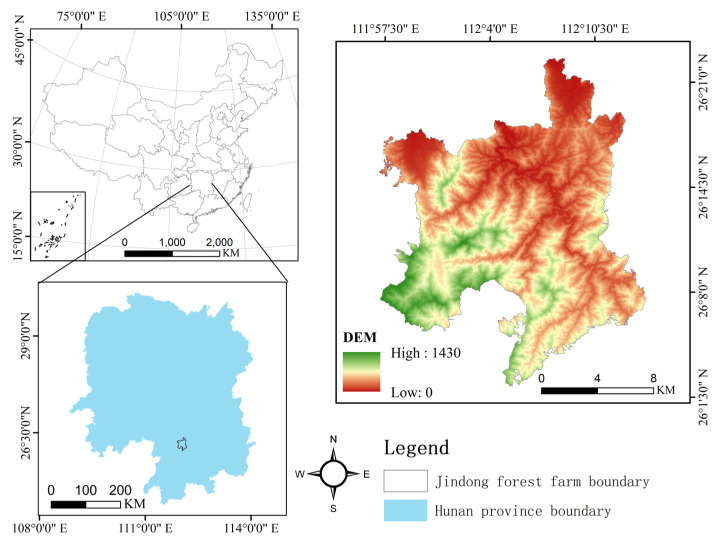
Location of the study site.

Jindong Forest Farm is located in the southern part of Qiyang County, Hunan Province, China (Figure 4). It is located in the middle and upper reaches of the Xiangjiang River Basin, with dense mountains and steep slopes, with an average slope of 34° and 95.2% of grade IV and above (26° or above). The highest altitude is 1435 m and the lowest is 108 m. The soil of the forest farms is mainly yellow-red and yellow. The thickness of the soil layer is generally more than 60 cm, the gravel content is about 20% to 30%, and the average soil organic matter content is more than 2%, with the highest value reaching 11%. It belongs to the subtropical southeast monsoon humid climate zone, with an average annual temperature of 18 °C, an extreme maximum temperature of 41 °C and an extreme minimum temperature of −8 °C. The average annual effective sunshine duration is 1617 h, the average annual precipitation is 1600–1890 mm, and the average annual evaporation is 1225 mm. The relative humidity is 75–82%, the annual frost-free period is 265–349 days, and the vegetation has 281–301 natural days [33]. There are 972 species belonging to 135 families in the forest farm. According to the survey, there are over 1500 species of higher plants belonging to more than 200 families. There are 654 species of woody plants in 98 families. At present, *Ginkgo biloba*, *Taxus chinensis* and others are first-class plants under state protection. The second-class protected plants are *Cinnamomum bodinieri*, *Pseudotsuga sinensis*, *Fokienia hodginsii*, *Phoebe bournei*, *Eucommia ulmoides*, and others. There are more than 190 species of terrestrial vertebrates in this area, of which 31 species are in the category of National Key Protected Animals, such as *Syrmaticus ellioti*, *Moschus berezovskii*, *Neofelis nebulosa*, and others.

### 4.2. Data Collection

**Figure 5:**
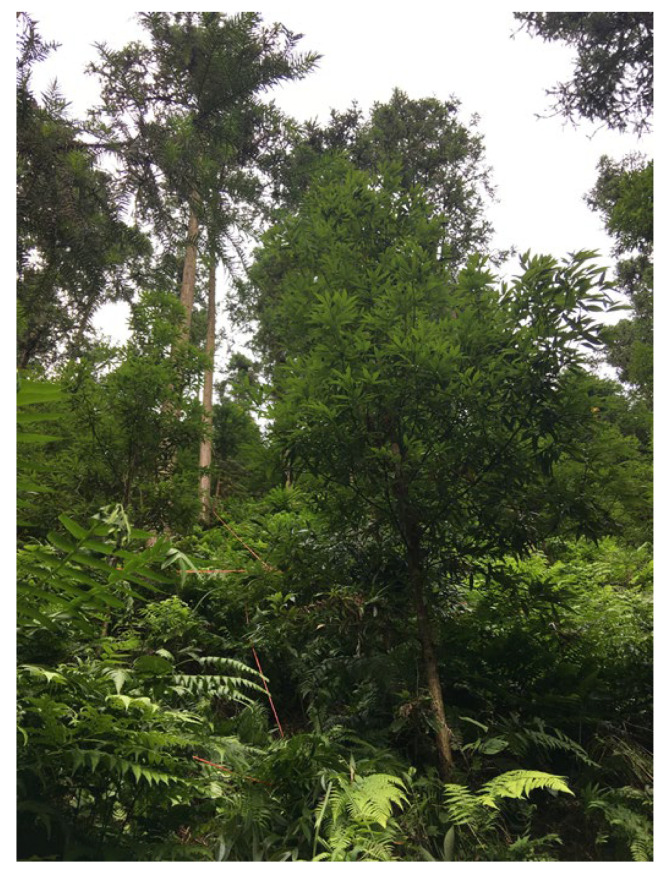
Our sample plots were replanted with *Phoebe bournei* in the understory of *Cunninghamia lanceolata* trees to create a mixed forest of *CLPB*, changing the stand structure to form a heterogeneous, complex, mixed forest.

**Figure 6:**
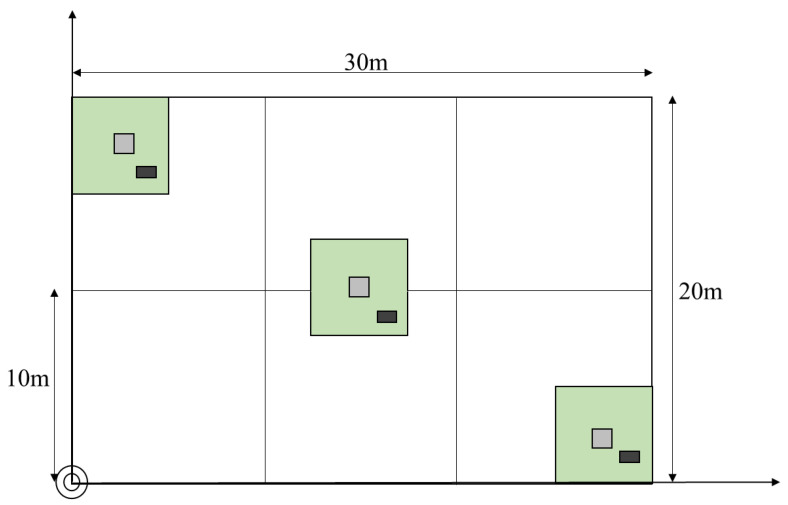
Survey units for trees, shrubs, herbs, and soils.

The field survey was conducted from July to August every year from 2015 to 2019. The sample plots were randomly selected from mixed forests of *CLPB* with the same stand phase and age, and were representative (Figure 5). A total of 40 plots (20 × 30 m) were monitored for five consecutive periods. Each sample plot was divided into 6 survey units (10 × 10 m), giving a total of 240 data points. According to the growth and dispersion characteristics of undergrowth vegetation, representative shrub quadrats (5 × 5 m) were set up in the upper, middle and lower sample plots of each plot, and one (1 × 1 m) herb quadrat was set in each shrub quadrat. A total of 120 shrub quadrats and 120 herbaceous quadrats were established in this study (Figure 6). Three soil profiles with a width of 0.8~1.0 m and a depth of 0.6~0.8 m were evenly set in each standard land area, and four samples from layers 0~15 cm, 15~30 cm, 30~45 cm and 45~60 cm deep were obtained with a ring knife, each with a volume of 100 cm^3^. Three soil samples were taken from each layer to determine their physical properties.

All trees in the plots with a diameter at breast height (DBH) larger than 5 cm were investigated. Starting from the lower-left corner of each plot, trees in the plot were numbered according to an “S” shape. For each tree, the tree species, tree height (m), DBH (cm) and position were recorded. The species, number, height (m), and coverage of shrubs (%) and herbs in each plot were recorded. The surveyed information of the plots also included altitude (m), terrain, slope (°), position, aspect, and soil type.

### 4.3. Data Processing

After collecting preliminary data for the forest and land samples, we proceeded to analyze this data to obtain multifunctional index values for the forest stands.

#### 4.3.1. Productivity Measurement

In this study, the index of the stand’s productivity was denoted by the live wood stock. The storage capacity of living trees was calculated by the binary volume formula (Table 2) and converted into the storage amount per unit area.

#### 4.3.2. Carbon Sequestration of Vegetation and Soil

Forest carbon comprises vegetation and soil carbon reserves. In this study, vegetation carbon was subdivided into tree-layer carbon, undergrowth-layer, shrub-layer and herb-layer carbon and litter-layer carbon. The tree-layer carbon was calculated using the forest biomass allometric growth equation [46,47,48,49,50], while we used the biomass method to estimate forest carbon at all levels (Table 3).

Biomass was measured in the shrub, herb and litter layers using the quadrat all-harvest method. Fresh weights were weighed immediately after harvesting. After returning to the laboratory, the dry biomass was baked at 105 °C for 6–8 h to a constant weight, and the dry biomass was calculated and converted into biomass per unit area. The conversion methods of carbon storage and biomass are as follows:

(1) $$C=BC_{C}$$

where *C* represents carbon storage (*t*); *B* stands for forest biomass (*t*); and *C_c_* represents the carbon content, where the carbon conversion coefficient is 0.5 for coniferous forest and 0.45 for broadleaved forest.

Soil carbon storage is represented by the product of soil bulk density, soil thickness and soil organic carbon content. The calculation formula is as follows:

(2) TOC=θ·D·C

where *TOC* represents soil carbon storage (t/hm^2^), *θ* represents soil bulk density (g/cm^3^), *D* represents soil thickness (cm), and *C* represents soil organic carbon content (%).

#### 4.3.3. Species Diversity

The relative abundance, relative cover (relative significance) and relative frequency of understory vegetation were calculated to represent the important values (IV) of different shrub and herb species in the plots. The species diversity index was calculated based on the species’ importance values. In this paper, we use the Patrick richness index (*D*), Shannon–Wiener diversity index (*H*), Simpson dominance index (*H’*) and Pielou uniformity index (*J*) to comprehensively evaluate the species diversity of a community, which is calculated as follows:

Ⅳ = (Relative frequency + relative coverage + relative abundance)/3 × 100%(3)

(4) $$H=-\overset{s}{\underset{i=1}{\sum }}p_{i}lnp_{i}$$

(5) $$J=\left(-\overset{s}{\underset{i=1}{\sum }}p_{i}lnp_{i}\right)/lnS$$

(6) $$H^{\prime }=1-\sum_{i=1}^{s}p_{i}^{2}$$

*D* = *S* (7)

where *p_i_* is the ratio of the number of individuals of the *i* species to the total number of individuals, and *S* refers to the total number of species in the sample.

This study uses survey units (10 × 10 m) as data points, so all data refer to the average of one survey unit. All statistical analyses were performed using SPSS 20.0 for Windows, and the SEM was constructed with Amos 22.0 software.

### 4.4. Structural Equation Model

The structural equation model consists of a measurement model and a structural model [24,51,52]. SEM can analyze the causal relationships between multiple variables in a system and clearly determine the relative importance of each relationship. The model can test the relationship between the explicit variables, latent variables, and error variables of the data, and then obtain the total, direct and indirect effects of the independent variables on the dependent variables [53,54,55].

**Figure 7:**
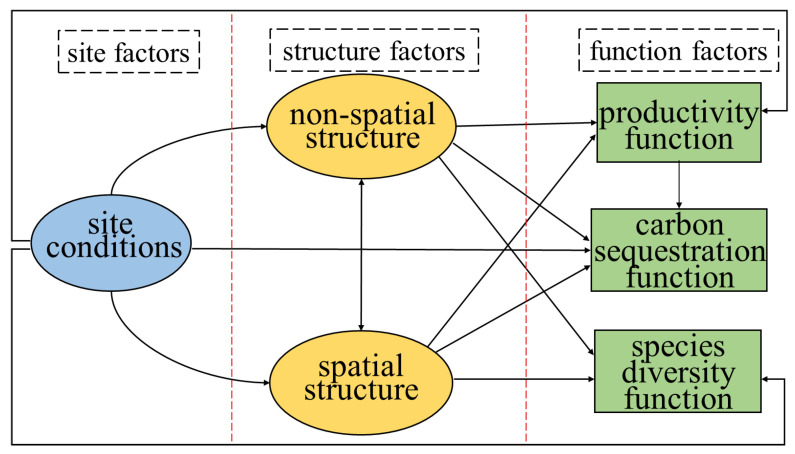
The a priori model based on the hypothesis relating stand structures, site conditions and stand functions. Structure factors include non-spatial structure and spatial structure; function factors include productivity, carbon sequestration and species diversity functions.

Based on the field survey as well as the theoretical and experimental studies con-ducted in these forest ecosystems [56,57,58,59], we established an initial SEM to assess the relative importance of structure variables (spatial structure and non-spatial structure) and site characteristics as the drivers of the response variables to forest functions. In the SEM, three latent variables were established, namely site conditions (determined by observed variables of slope position, dominant tree breast diameter and dominant tree height), spatial structure (determined by observed variables of dominance, mingling and uniform angle index), and non-spatial structure (determined by observed variables of DBH, tree height, and stand density). To test whether these indicators related to the functions of the forest area, Pearson correlation coefficients were calculated before establishing the SEM. The indicators showing significant relationship effects were selected for constructing the SEM. We built an initial model based on the a priori hypothesis (Figure 7), and the model was tested using the goodness of fit index (GFI), comparative fit index (CFI), and root mean square error of approximation (RMSEA) [60,61]. The optimal model should have the lowest RMSEA value < 0.05, and the highest GFI value and CFI value > 0.90.

## 5. Conclusions

In this study, we used the SEM to quantify the relative importance of the stand structure and site conditions of *CLPB* to productivity, species diversity and carbon sequestration in Jindong Forest Farm, Hunan Province. Using data from 40 plots, with 240 survey units, we found that site conditions have a greater impact on forest function than stand structure. In addition, compared to spatial structures, non-spatial structures have a greater overall impact on forest functions. The results demonstrated that the close-to-natural forest management practices should be prioritized for pure Chinese fir forests with better site conditions, which can improve the ecological function of pure Chinese fir forests more effectively. In addition, adjusting the stand structure of Chinese fir forests can substantially improve the ecological performance.

## Figures and Tables

**Table 1:** Fitting parameters of structural equation model.

Statistics	Fitting Index	Evaluation Standard	A Priori Model	Optimal Model
Absolute fit statistics	$\chi^{2}/df$	Between 1–3 means the model fits well	1.889	1.889
	GFI	>0.90	0.949	0.935
	RMSEA	<0.05	0.055	0.038
	NCP	The smaller the better	39.995	28.367
Value-added adaptation statistics	NFI	Between 0–1, the closer to 1, the better the model fit	0.948	0.954
	RFI		0.931	0.933
	IFI		0.975	0.978
	TLI		0.962	0.967
	CFI		0.974	0.978
Minimal adaptation statistics	PGFI	>0.5, The higher the value, the better	0.548	0.550
	PNFI	>0.5, The higher the value, the better	0.649	0.651

**Table 2:** Two-dimensional volume table of main trees.

Species	Formula	a	b	c
*Cunninghamia lanceolata*	*V* = a × *D*^b^ × *H*^c^	0.000058777042	1.969983	0.896462
Other conifer	*V* = a × *D*^b^ × *H*^c^	0.000062341803	1.855150	0.956825
One type of hardwood broad-leaf	*V* = a × *D*^b^ × *H*^c^	0.000068563400	1.933221	0.867885
Second type of hardwood broad-leaf	*V* = a × *D*^b^ × *H*^c^	0.000050479055	1.88452	0.990765
Soft broad-leaf	*V* = a × *D*^b^ × *H*^c^	0.000041028005	1.80063	1.130599

Note: *D* is DBH (cm), *H* is tree height (m). *Phoebe bournei* is a type of hardwood broad-leaf tree.

**Table 3:** Biomass allometric growth equation of different tree species in the tree layer.

Species	Biomass Equation	R^2^
*Cunninghamia lanceolata*	*W*_trunk_ = 37.9323*D*^2.598^	0.975
	*W*_branch_ = 1.6255*D*^2.0074^	0.764
	*W*_leaf_ = 5.2619*D*^2.1515^	0.788
	lg*W*_root_ = −1.995 + 2.4541lg*D*	0.962
Hard broad-leaf class	*W*_trunk_ = 0.065*D*^2.548^	0.972
	*W*_branch_ = 0.025*D*^2.390^	0.91
	*W*_leaf_ = 0.036*D*^1.818^	0.876
	*W*_root_ = 0.027*D*^2.394^	0.922
Soft broad-leaf class	*W*_trunk_ = 0.080*D*^2.348^	0.995
	*W*_branch_ = 0.027*D*^1.762^	0.975
	*W*_leaf_ = 0.027*D*^1.371^	0.954
	*W*_root_ = 0.027*D*^2.165^	0.873

Note: *D* is DBH (cm). See references [46,47,48,49,50].

## Data Availability

The data from the sample plots in this study are available on request from the corresponding author. Those data are not publicly available due to privacy and confidentiality.
